# *Bacillus velezensis* A2 fermentation exerts a protective effect on renal injury induced by Zearalenone in mice

**DOI:** 10.1038/s41598-018-32006-z

**Published:** 2018-09-11

**Authors:** Nan Wang, Peng Li, Jiawen Pan, Mingyang Wang, Miao Long, Jian Zang, Shuhua Yang

**Affiliations:** 1 Key Laboratory of Zoonosis of Liaoning Province, College of Animal Science & Veterinary Medicine, Shenyang Agricultural University, Shenyang, 110866 China; 2 Testing& Analysis Center, Shenyang Agricultural University, Shenyang, 110866 China

## Abstract

Zearalenone (ZEN) is an estrogen-like mycotoxin occurring in food and feeds, and it can cause oxidative damage and apoptosis in the testis, liver, and kidney. A current concern for researchers is how to reduce the harm it causes to humans and animals. In this study, our aim was to isolate and identify a novel and efficient ZEN-detoxifying strain of bacteria, and we aimed to assess the protective effect of the isolated strain on kidney damage caused by ZEN in mice. Our results indicated that a strain of *Bacillus velezensis* (*B*. *velezensis*), named A2, could completely degrade ZEN (7.45 μg/mL) after three days of incubation at 37 °C in the Luria-Bertani (LB) medium. This fermentation broth of the *B*. *velezensis* A2 strain was given to mice. The histopathological analysis indicated that the fermentation broth from the *B*. *velezensis* A2 strain reduced the degree of renal injury that is induced by ZEN. Furthermore, it greatly reduced the increase in serum levels of creatinine (CRE), uric acid (UA), and urea nitrogen (BUN) caused by ZEN. In addition, *B*. *velezensis* A2 strain also significantly inhibited the increase of malonaldehyde (MDA) content, and reversed the decreases of total superoxide dismutase (T-SOD) and glutathione peroxidase (GSH-Px) activities caused by ZEN. Studies have shown that ZEN is involved in the regulation of mRNA and protein levels of genes involved in the ER stress-induced apoptotic pathway, such as heavy chain binding protein (BIP), C-/-EBP homologous protein (CHOP), cysteine Aspartate-specific protease-12 (Caspase-12), c-Jun N-terminal kinase (JNK), and BCL2-related X protein (Bcl-2 and Bax). However, when mice were administered the fermentation broth of the *B*. *velezensis* A2 strain, it significantly reversed the expressions of these genes in their kidney tissue. In conclusion, our results indicate that the newly identified strain of *B*. *velezensis* A2, has a protective effect from renal injury induced by ZEN in mice. This strain has a potential application in the detoxification of ZEN in feed and protects animals from ZEN poisoning.

## Introduction

Zearalenone is a well-known F2 toxin that is produced by *Fusarium fungi*, and it is commonly found in moldy grain^1–3^. Contamination of ZEN in cereals is an ongoing global concern^4–6^. ZEN has harmed animals through food enrichment with cereals, and then it affects human health through the food chain^7–9^. ZEN is known to induce cytotoxicity and oxidative damage, and its toxicity primarily manifests in the reproductive system^10–12^, the liver^13,14^, and the kidneys^15,16^. ZEN binds to the estrogen receptor in the cytoplasm; this causes lipid peroxidation and a series of cytotoxic effects that alter serum enzymology and ROS levels^17,18^. Many studies have shown that ZEN is highly toxic to the blood, kidneys, and liver of mice. Additionally, ZEN could alter the enzymatic and hematological parameters of mice, and it could induce higher levels of oxidative stress^19–21^. Meanwhile, ZEN-induced apoptosis is characterized by ROS production and increased lipid peroxidation^22,23^. A previous study demonstrated that ZEN induced apoptosis in RAW 264.7 macrophages through endoplasmic reticulum stress (ER stress)^24^.

Biological detoxification is an optimal method for ZEN detoxification because it has the advantages of high specificity, high efficiency, and non-toxic metabolites. ZEN can be degraded by several strains. Previous studies have shown that bio-detoxification agents can transform ZEN into less toxic or non-toxic metabolites. These bio-detoxification agents include *B*. *subtilis*^25,26^, *B*. *amyloliquefaciens*^27^, *B*. *licheniformis*^28,29^, *Rhodococcus* K408^30^, *L*. *rhamnosus*^31^, *L*. *plantarum*^32^, *L*. *reuteri*^33^, and *S*. *cerevisiae*^34,35^. These strains provide a mycotoxin detoxification strategy for cell wall adsorption and activate enzyme degradation pathways. However, these strains cannot completely eliminate ZEN during short-term fermentation. Our study found that the A2 strain completely removed ZEN (7.45 μg/mL) after three days of fermentation, which was not reported for the other strains. Our research supports a viable detoxification program for the control of mycotoxins in the future.

Reports have shown that *B*. *licheniformis* CK1 degrades ZEN in feed and alleviates the toxicity of ZEN in piglets^29^. Previous research has shown that β-glucan in the cell wall of *yeast* has the ability to adsorb ZEN. This may be why feeding the *yeast* products had the ability to inhibit the effects of ZEN on the growth and health of pigs^35–37^. It has also been reported that ZEN-contaminated Luria-Bertani (LB) broth does not exhibit the endocrine disrupting effects induced by ZEN in rats treated with *R*. *pyridinivorans* K408^30^. In ZEN detoxification studies, the impact of probiotic strains has also been reported on distal organs in animals. For example, treatment with *L*. *paracasei* BEJ01 reduceds the immunotoxic effects that were induced by ZEN^38^. *L*. *rhamnosus* GG prevented or treated the damaging effects of ZEN in mice by regulating the secretion of mucus by goblet cells and improving plasma D-lactate, serum IL-8, and immunoglobulin (Ig) levels^31^.

In the present study, we aimed to identify a new ZEN-degrading strain and to determine its detoxification effects *in vitro*. Then, we aimed to evaluate the protective effects of the isolated strain on kidney damage caused by ZEN in mice. This study could possibly lay a foundation for the detoxification of ZEN and the efficient biotransformation of ZEN degradation with probiotics. The A2 strain was isolated from soil, and it demonstrated a high capacity to degrade ZEN. To better assess the ability of the A2 strain to degrade ZEN, we conducted *in vitro* and *in vivo* tests that identify strains and determine detoxification effects. The safety of the strains was verified in the animal experiments, and this could lay a foundation for future production practice.

## Results

### Strain screening

**Figure 1:**
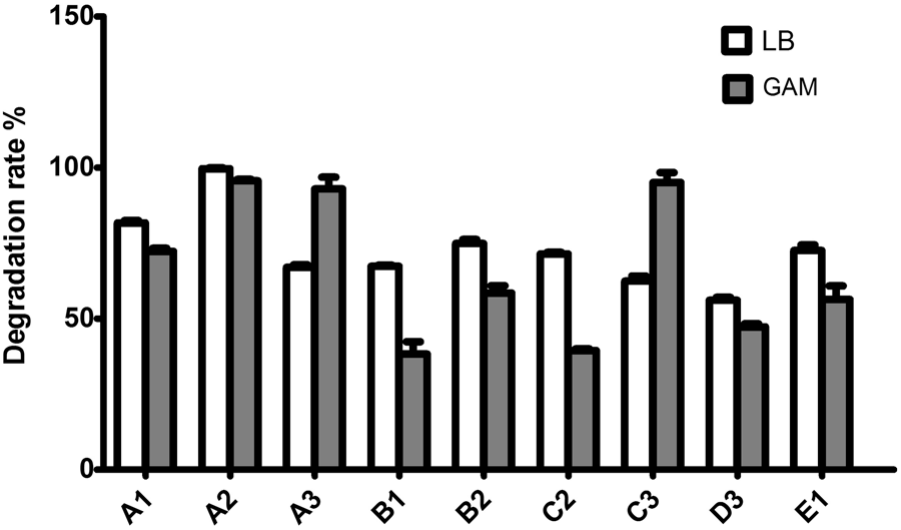
Isolation of ZEN degrading strains. A total of 9 strains were incubated with ZEN in GAM medium (**A**) or LB medium (**B**) for 72 h. Their detoxifying rates are shown as a percentage. The white column indicates A2 incubated in LB medium, and the gray column indicates A2 incubated in GAM medium.

After separation and enrichment, the bacterial strains isolated from soil were examined for their ZEN-degrading capabilities (Fig. 1). In gifu anaerobic medium (GAM), the strains A1, A2, A3, B2, C3, and E1 demonstrated the highest degrading capability. Their detoxifying rates were 74.07%, 94.68%, 100.00%, 56.60%, 100.00%, and 52.08% of total zearalenone after 72 h, respectively. In Luria-Bertani (LB) medium, the strains A1, A2, A3, B1, B2, C2, C3, D3, and E1 demonstrated the highest degrading capability. Their detoxifying rates were 80.74%, 100.00%, 66.67%, 67.90%, 76.89%, 71.60%, 65.43%, 58.02%, and 72.84% of total zearalenone after 72 h, respectively. The same strain demonstrated different rates of degradation in different media. This could be due to differences in the degradation rate of ZEN under the conditions of different nutrient components, such as the carbon source and nitrogen source in the different media. The optimal degradation rate was observed in the A2 strain. The A2 strain exhibited a strong degradation capacity of ZEN. The ZEN-detoxifying rates for the A2 strain were 94.68% and 100.00% in GAM and LB medium, respectively. Therefore, we used the A2 strain as a ZEN antidote for subsequent experiments.

### Identification of the A2 strain

**Figure 2:**
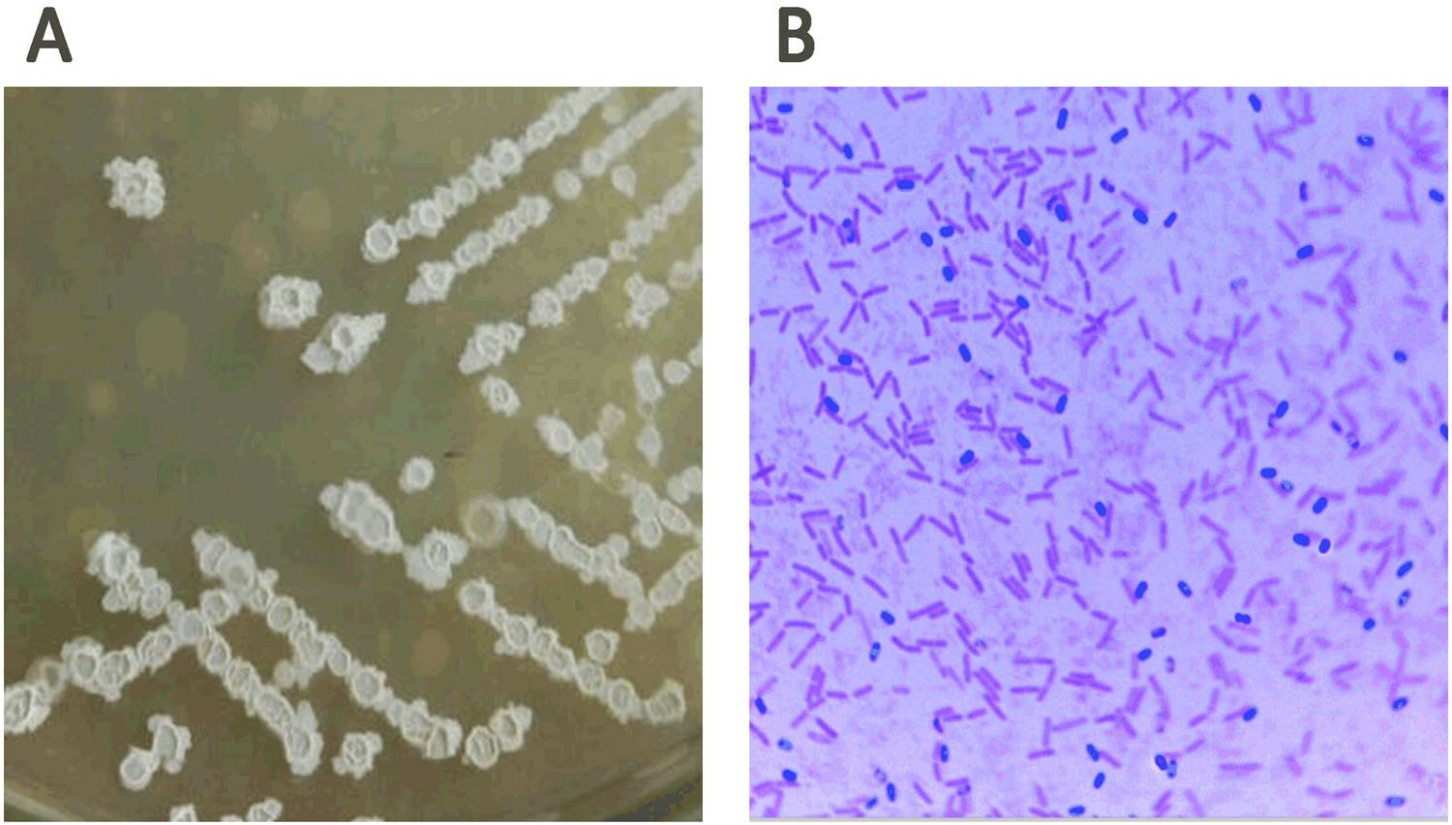
Colony morphology and spore staining morphology of the A2 strain grown on an LB culture plate. (**A**) The A2 strain was inoculated on an LB agar plate and cultured at 37 °C for 24 h. (**B**) The A2 strain was inoculated into LB medium at 37 °C, and it was shaken at 150 rpm for 36 h to prepare spores. Spores were stained green with 5% malachite green. The A2 strains were stained red with 5% Safranin O, which are in the form of short rods.

**Figure 3:**
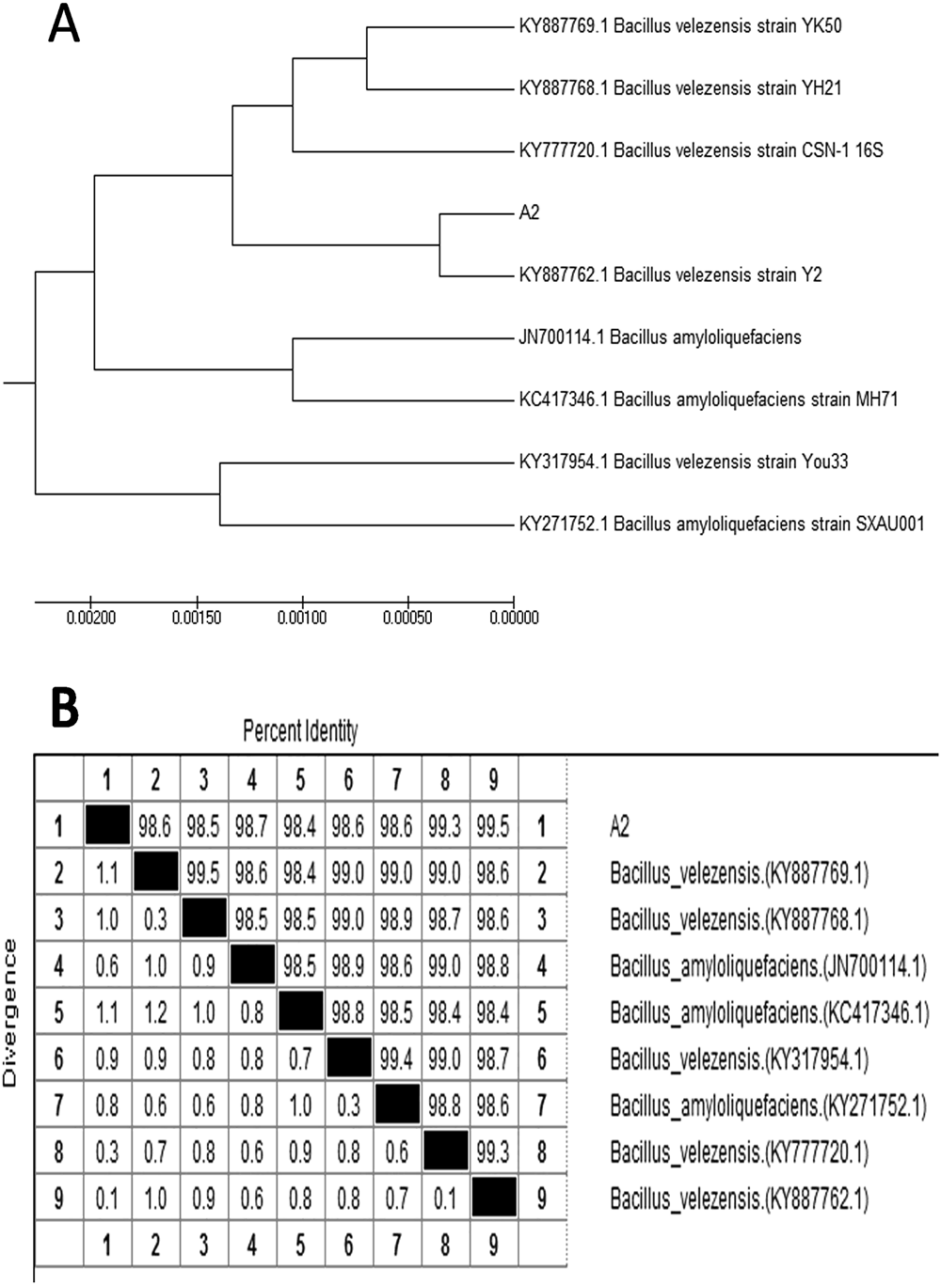
Homology anlysis based on partial 16 S rDNA sequences of the A2 strain and the related microorganisms. (**A**) The phylogenetic tree was made by MEGA7. The trees were constructed by the maximum likelihood method. The GenBank accession numbers are listed in the front of the bacteria strain name. The A2 strain (GenBank: MG727659.1) is located on the *Bacillus velezensis* branch. (**B**) Homology analysis was performed by ClustalW. The 16 S rDNA sequence of the A2 strain (1452 bp) is 99.5% identical to *Bacillus velezensis* Y2 (GenBank: KY887762.1). The number in parentheses is the GenBank accession number of the sequence.

The A2 strain grew normally and in accordance with the close-packed rule for culture streaking. The colonies of this strain were white, skin-like pellicles that were slimy with a ridged surface when grown in LB (Fig. 2A). It was a Gram-positive bacillus strain, and a large number of spores were produced when cultured at 37 °C for 36 h in LB medium (Fig. 2B). After constructing the phylogenic tree, the Gram-positive A2 strain was located on the *B*. *velezensis* branch (Fig. 3A). Additionally, the A2 strain shared 99.5% similarity with *B*. *velezensis* Y2 (Fig. 3B). Thus, we concluded that the A2 strain is a member of the *B*. *velezensis* species.

### Zearalenone degradation

**Figure 4:**
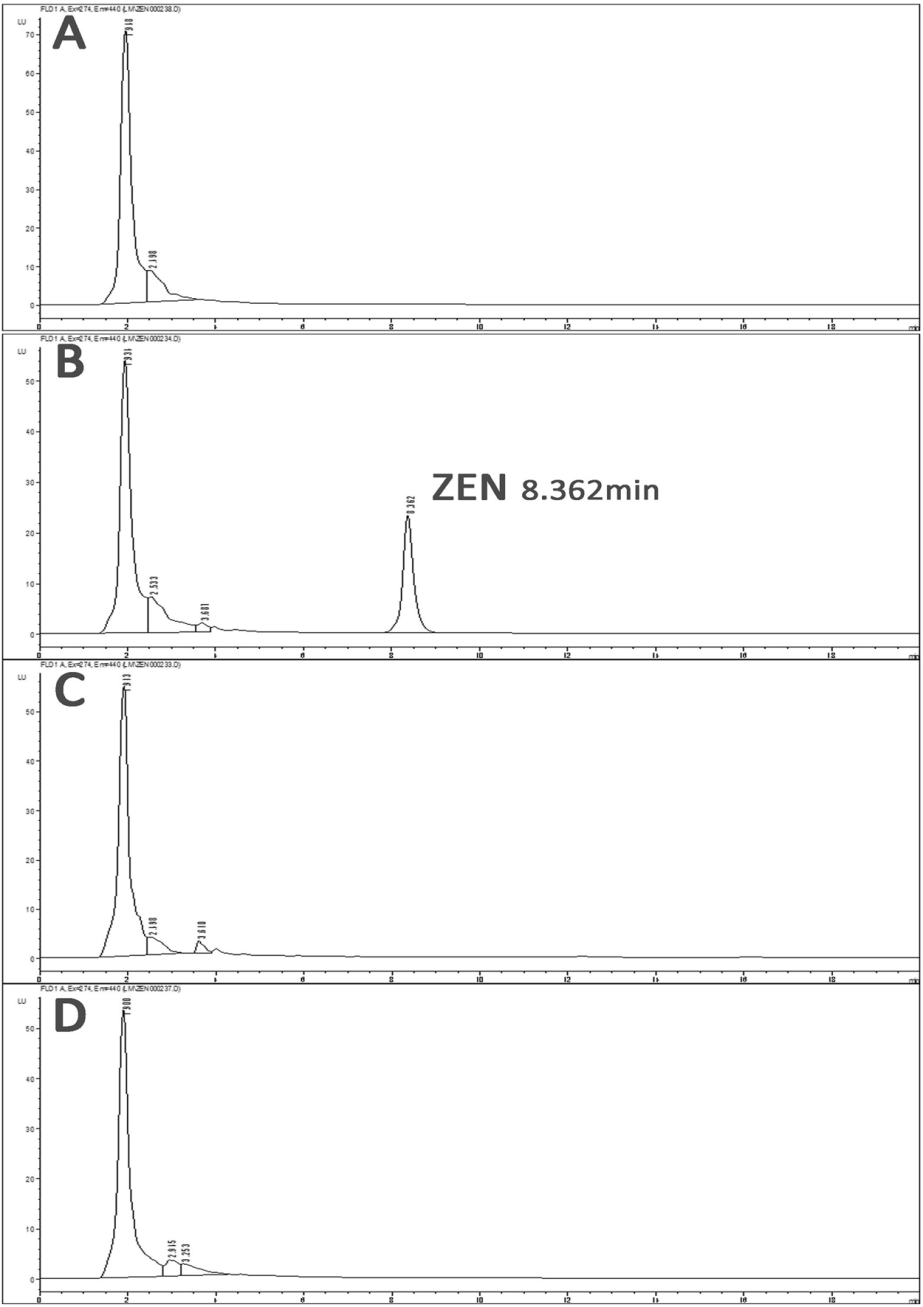
The A2 strain was incubated with ZEN (7.45 μg/mL) in LB medium for 72 h, and the residual amount of ZEN was determined by HPLC. (**A**) Uncontaminated LB medium (control group). (**B**) LB medium containing ZEN (7.45 μg/mL) (ZEN group). (**C**) LB medium containing only the A2 strain (A2 group). (**D**) ZEN-containing LB medium after A2 strain treatment (A2 + ZEN group).

Based on the HPLC peak of the control group, in the ZEN group, HPLC revealed a material peak around 8 minutes in the ZEN group (Fig. 4A,B). However, in the A2 group and the A2 + ZEN group, this peak was not observed (Fig. 4C,D). These results indicate that the A2 strain was fermented in LB medium for 72 h, and it completely eliminated the ZEN toxin (7.45 μg/mL).

### Histopathological changes in the kidney

**Figure 5:**
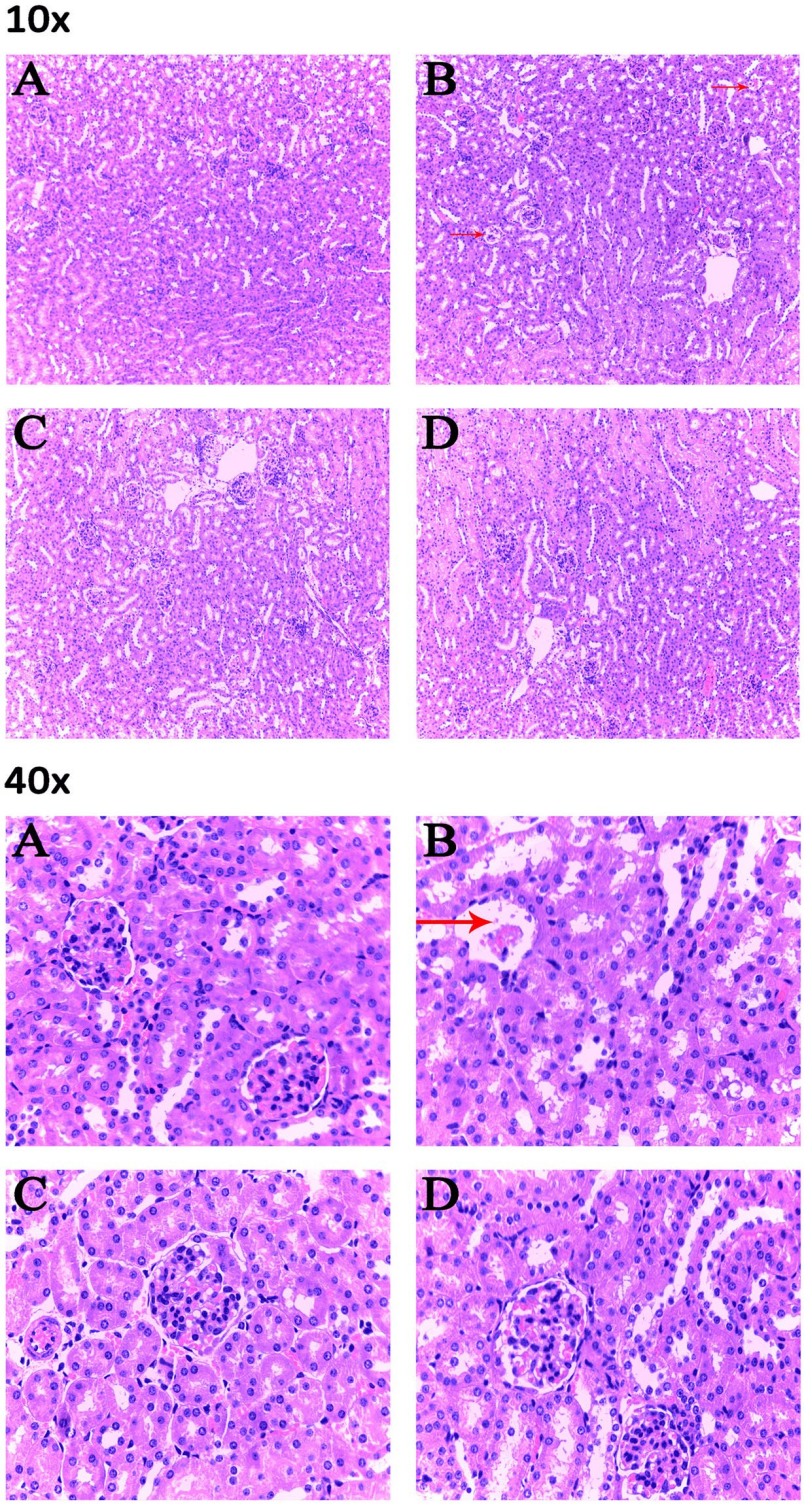
Detection of pathological changes in renal tissue by tissue section HE staining. Images were taken at a magnification of X10 and X40. (**A**) Control group, (**B**) ZEN group, (**C**) A2 group, and (**D**) A2 + ZEN group. The arrows “ → ” indicate a pathological injury in the kidney, such as glomerulus lobulation and glomerulus atrophy.

*In vivo* results show that ZEN caused lobulation and atrophy of the glomerulus in murine kidneys (Fig. 5B). Furthermore, the kidney sections from the A2 and the A2 + ZEN groups revealed minor pathomorphological changes (Fig. 5C,D). These results indicate that co-administration of the A2 fermentation broth exerted a protective effect from ZEN-induced kidney injury.

### Organ coefficient and blood biochemistry

**Figure 6:**
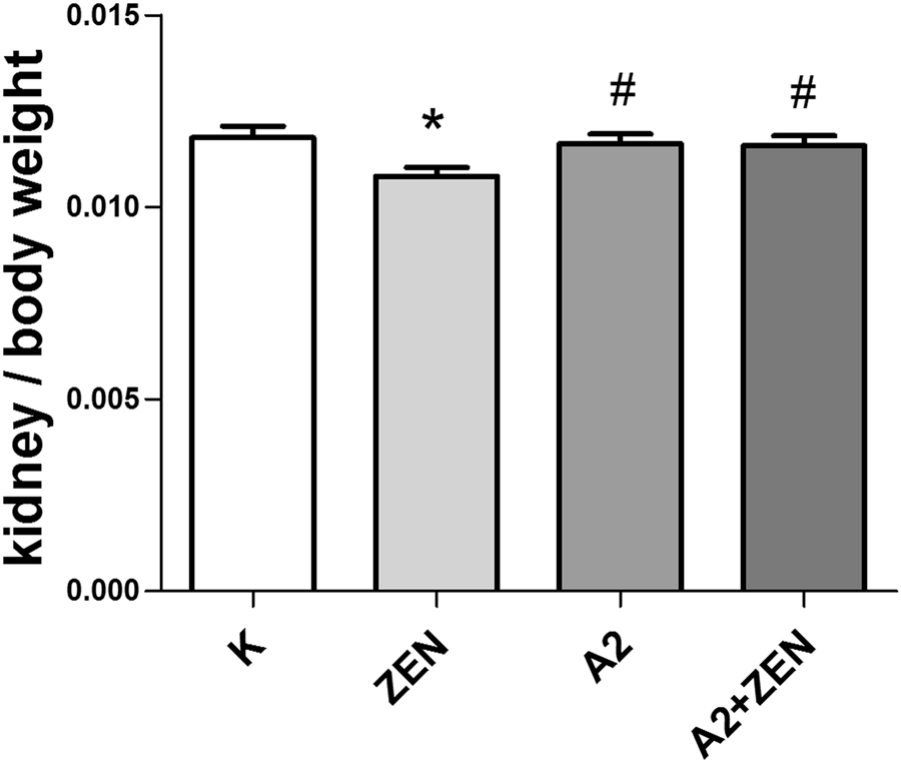
Observed changes in the ratio of kidney weight to body weight in mice. “*” Indicates significant differences compared to the control group (P < 0.05). “#” Iindicates significant differences compared to the ZEN group (P < 0.05).

**Figure 7:**
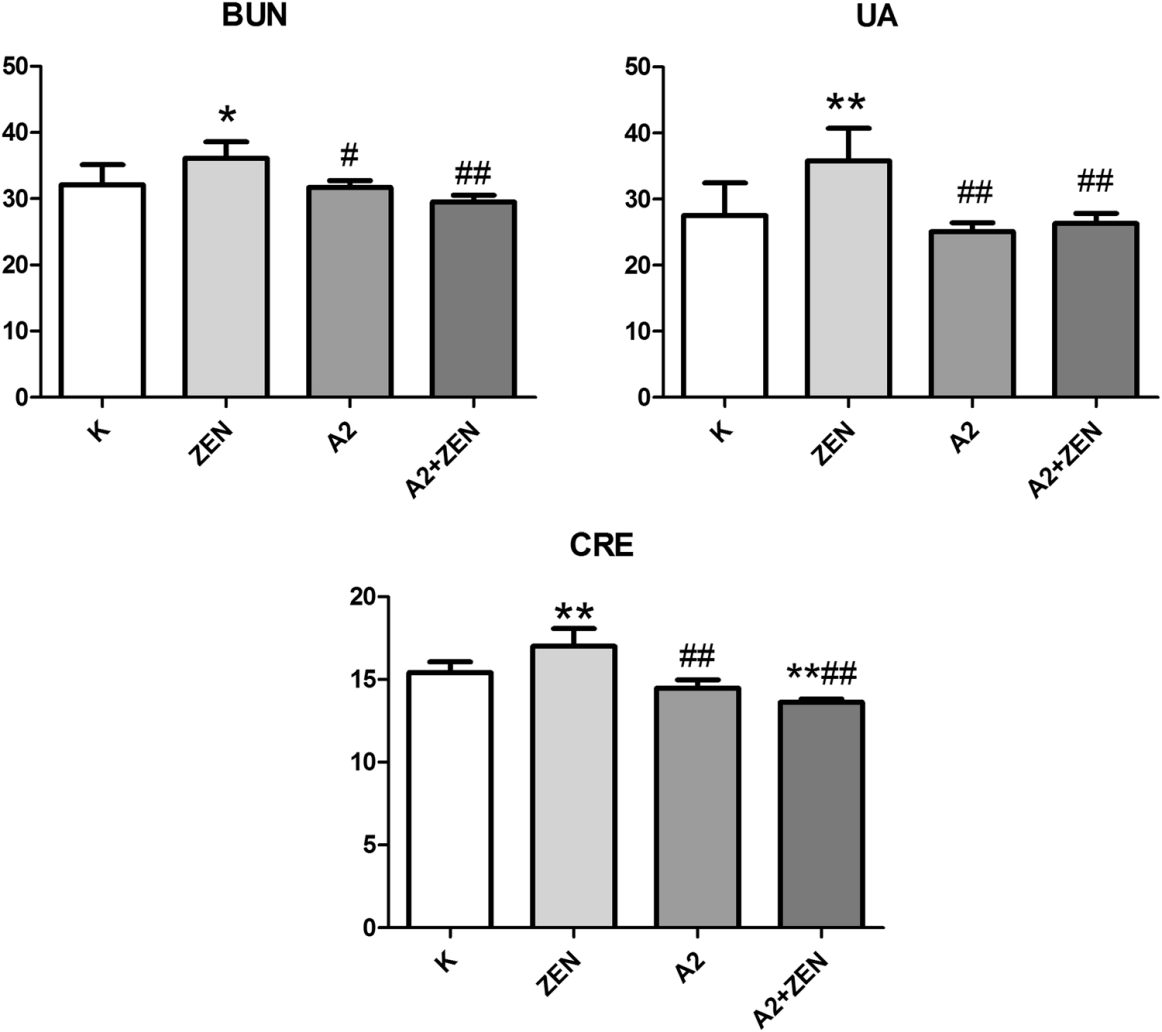
Serum enzymatic changes in each group of mice as determined by a serum test kit. “*” Indicates significant differences (*P* < 0.05) compared to the control group, and “**” indicates highly significant differences (*P* < 0.01) compared to the control group. “#” Indicates significant differences (*P* < 0.05) compared to the ZEN group, and “##” indicates highly significant differences (*P* < 0.01) compared to the ZEN group.

Our results show that the kidney indexes in the ZEN group were significantly decreased (P < 0.05). However, the kidney indexes were improved in the A2 + ZEN group compared to the ZEN group (P < 0.05) (Fig. 6). Serum activities of BUN, UA, and CRE function as biochemical markers that indicate renal damage. As shown in Fig. 7, there was a marked increase in serum BUN, UA, and CRE in the ZEN group (*p* < 0.05). When compared to the control group, there were no significant differences found in the A2 group and the A2 + ZEN group. Meanwhile, serum BUN, UA, and CRE were significantly reduced in the A2 + ZEN group, when compared to the ZEN group (P < 0.01). These results indicate that mice administered the A2 fermentation broth exerted a protective effect from ZEN-induced kidney injury.

### Antioxidant changes in the kidney

**Figure 8:**
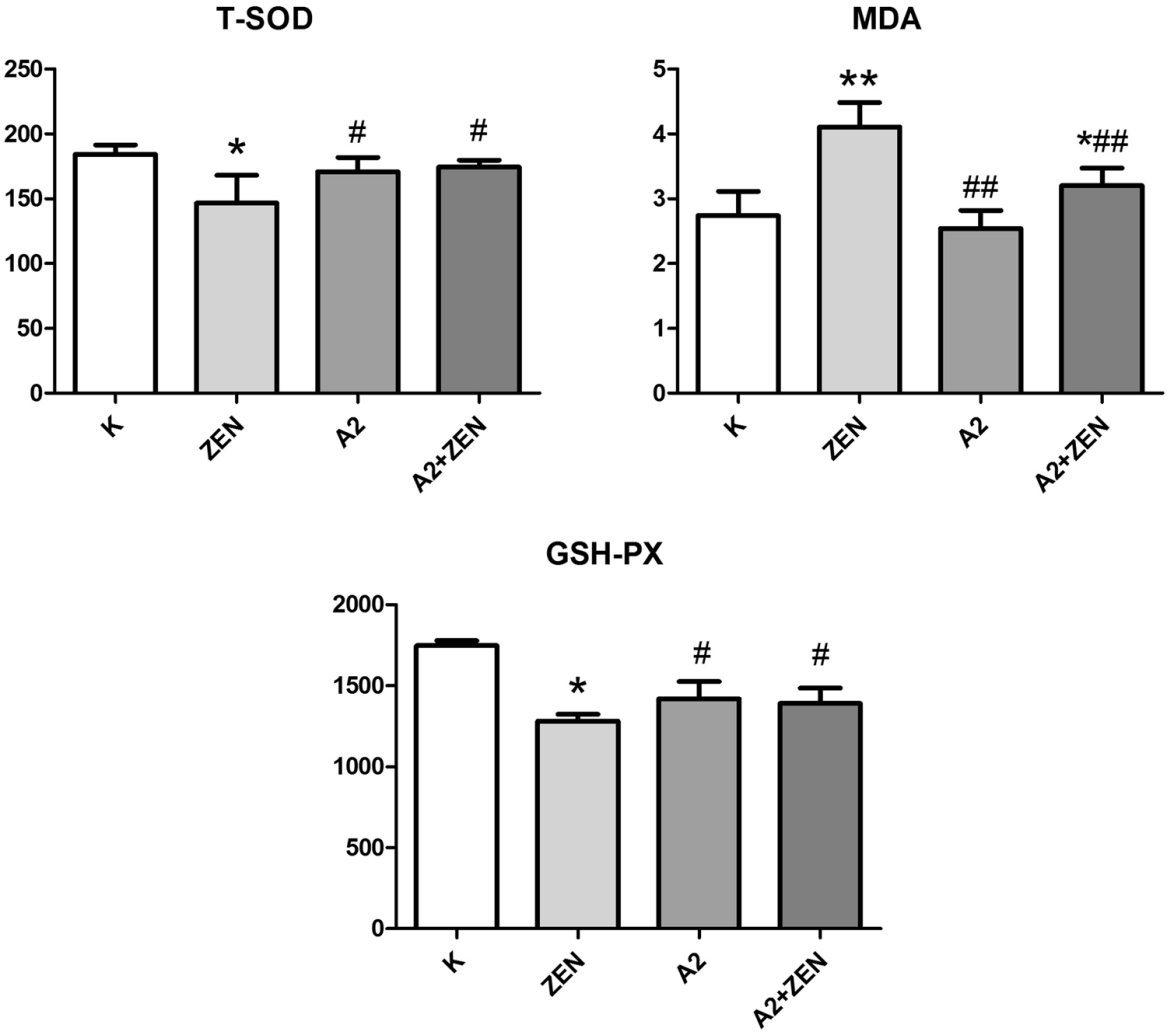
Oxidation and antioxidation parameters of renal tissues of mice in each group were detected by an oxidation kit. “*” Indicates significant differences (P < 0.05) compared to the control group, and “**” indicates highly significant differences (P < 0.01) compared to the control group. “#” Indicates significant differences (P < 0.05) compared to the ZEN group, and “##” indicates highly significant differences (P < 0.01) compared to the ZEN group.

The level of MDA in the kidney is used as an index to indicate lipid peroxidation caused by kidney damage. The levels of GSH-PX and T-SOD in the kidney are used as indexes to indicate antioxidants that reduce renal oxidative damage. As shown in Fig. 8, the levels of GSH-PX and T-SOD in the ZEN group were decreased (P < 0.05), and the MDA levels were significantly increased (P < 0.01) compared to the control group. However, oral administration of the *B*. *velezensis* A2 fermentation broth significantly inhibited the increase in MDA content caused by ZEN, and it reversed the decreases in T-SOD and GSH-Px activities caused by ZEN. These results indicate that co-administration of the A2 fermentation broth exerted a protective effect from ZEN-induced kidney injury.

### Gene and protein expression associated with the ER stress

**Figure 9:**
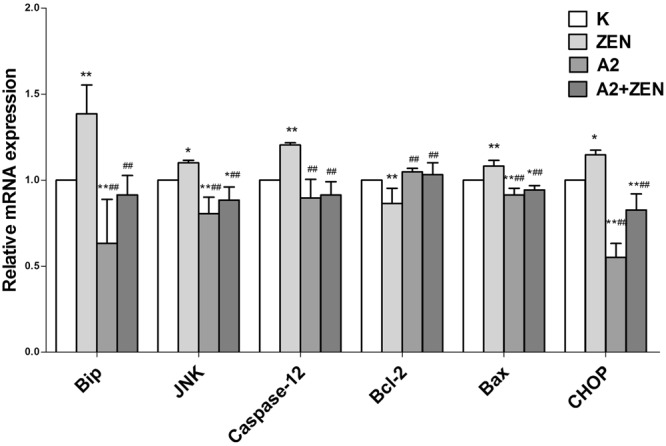
Effects of *B*. *velezensis* A2 fermentation broth on the expression levels of genes involved in the endoplasmic reticulum stress signaling pathway that was induced by ZEN in kidneys of mice. “*” Indicates significant differences (P < 0.05) compared to the control group, and “**” indicates highly significant differences (P < 0.01) compared to the control group. “#” Indicates significant differences (P < 0.05) compared to the ZEN group, and “##” indicates highly significant differences (P < 0.01) compared to the ZEN group.

**Figure 10:**
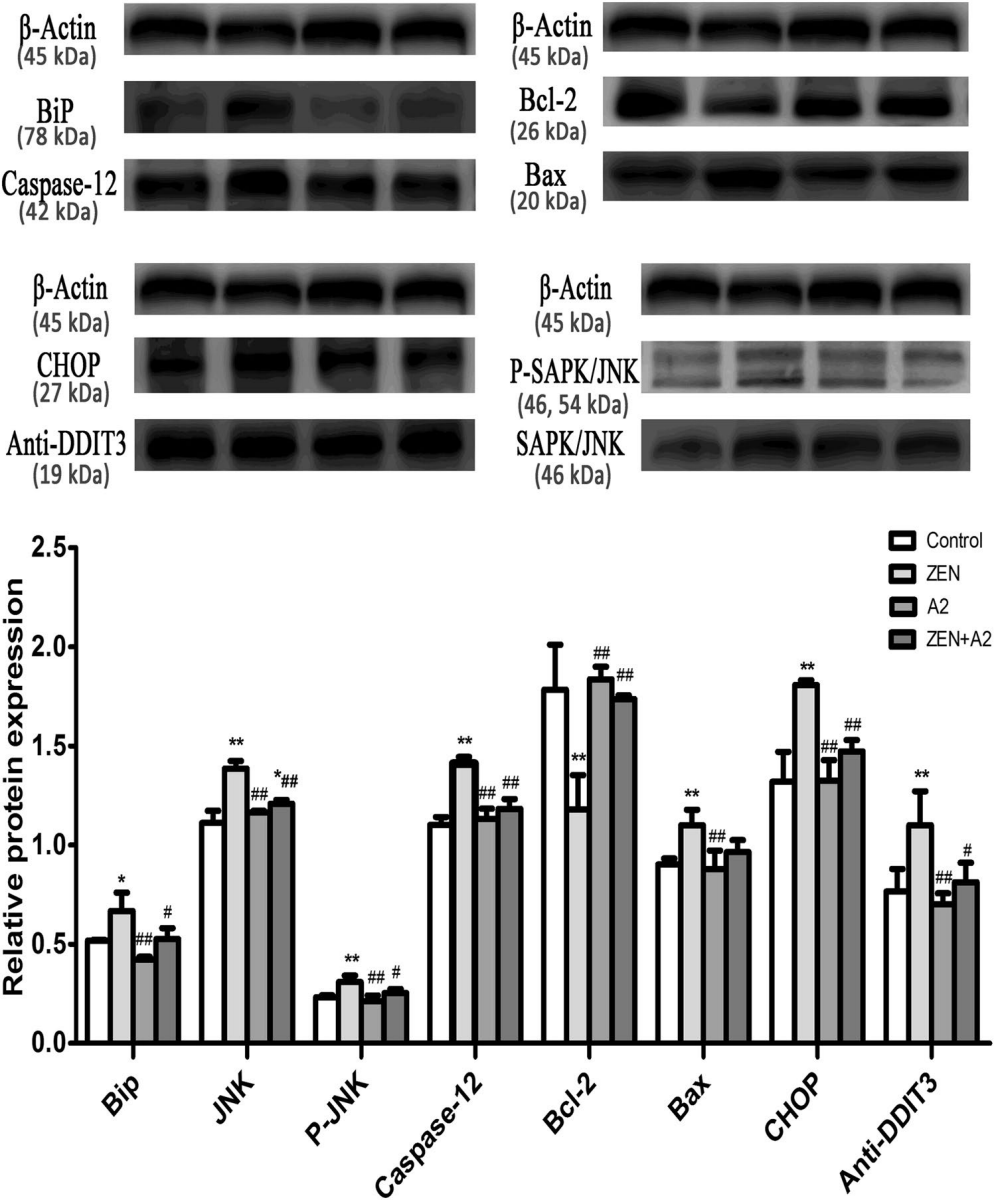
Effects of *B*. *velezensis* A2 fermentation broth on the expression levels of proteins involved in the endoplasmic reticulum stress signaling pathway that was induced by ZEN in the kidneys of mice. “*” Indicates significant differences (P < 0.05) compared to the control group, and “**” indicates highly significant differences (P < 0.01) compared to the control group. “#” Indicates significant differences (P < 0.05) compared to the ZEN group, and “##” indicates highly significant differences (P < 0.01) compared to the ZEN group.

**Figure 11:**
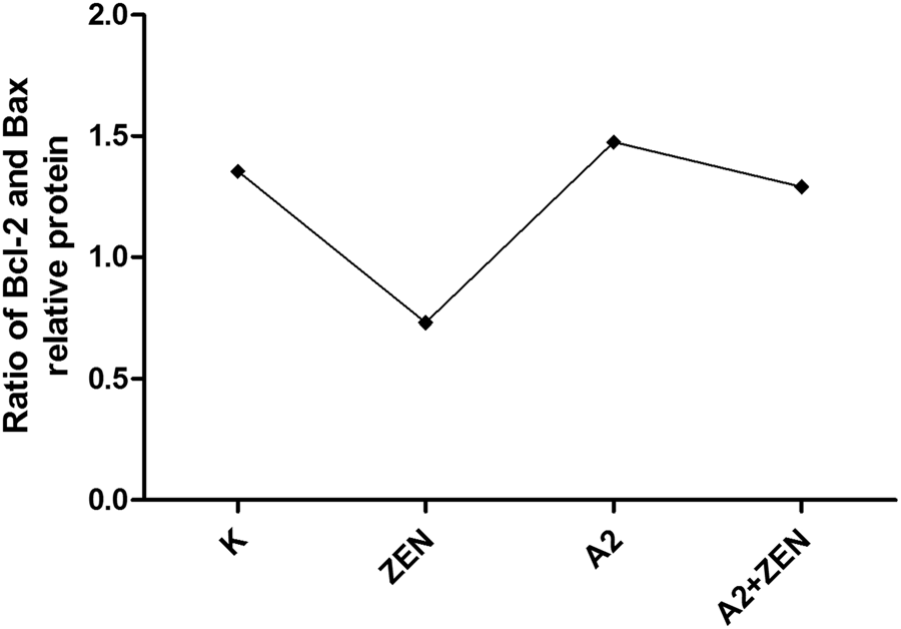
The ratio of Bcl-2 and Bax was used to indicate the apoptosis coefficient. (K) Control group, (ZEN) ZEN group, (A2) A2 group, and (A2 + ZEN) A2 + ZEN group.

The results of quantitative RT-PCR and Western blot analysis indicated that the A2 fermentation broth affects transcription factors that are associated with the ER stress signaling pathway (Figs 9, 10) (Supplementary Figure S1). When compared to the control group, there was a marked increase in the expression levels of BIP, JNK, Caspase-12, Bax, and CHOP in the ZEN group (P < 0.05). On the contrary, oral administration of the A2 fermentation broth greatly reduced the expression levels of BIP, JNK, Caspase-12, Bax, and CHOP (P < 0.05). Furthermore, Bcl-2 synthesis was down-regulated in the ZEN group (P < 0.01), and the co-administration of the A2 fermentation broth significantly reversed the decreased expression (P < 0.01). Overexpression of JNK and CHOP protein may induce protein phosphorylation and, ultimately, lead to cell apoptosis. As shown in Fig. 10, P-JNK and DDIT3 protein expression was detected in the ZEN group by Western blot analysis. The ratio of Bcl-2 and Bax has been proposed as a key factor in the regulation of apoptosis. As shown in Fig. 11, a low ratio of Bcl-2/Bax was observed in the ZEN group, and a low ratio of Bcl-2/Bax indicates increased apoptosis. However, there were no significant changes in the Bcl-2/Bax ratio between the A2 and A2 + ZEN groups. These results indicate that the A2 fermentation broth plays a regulatory role in ZEN-induced ER stress and apoptosis in murine kidneys.

## Discussion

In this study, we isolated and identified a *B*. *velezensis* strain, named A2, which demonstrated a high capacity to degrade ZEN. This strain has been deposited in the China Center for Type Culture Collection with the deposit number M2018352.

According to a previous study, following treatment of ZEN (5 μg/mL) and the *Rhodococcus* K408 strain for 5 days, HPLC analyses revealed a ZEN-degradation efficiency of 87.21%^30^. The isolated *B*. *subtilis* degraded more than 95% of the ZEN (1 mg/kg) when it was incubated in liquid medium for 24 h^26^. In addition, the study also demonstrated that ZEN was adsorbed by *yeast*^37^. However, this type of a degradation effect may not be enough to completely decontamination of ZEN. In this present study, a different ZEN-degradation efficiency was determined for the A2 strain of *B*. *velezensis*. When incubated in LB medium for 72 h, a 7.45 μg/mL dose of ZEN could be thoroughly degraded.

Previous research has shown that the *B*. *velezensis* strain can be used as a biocontrol agent for various plant diseases caused by phytopathogenic fungi, and it can effectively inhibit the growth of *Fusarium* by producing a variety of antimicrobial peptides^40–42^. However, a study on the application of *B*. *velezensis* as a method of mycotoxin detoxification has not been reported yet. In the current study, homologous sequence alignment showed a high level of homology between the *B*. *velezensis* A2 strain and *B*. *amyloliquefaciens*. In addition, *B*. *velezensis* is a biocontrol agent that exhibits broad spectrum antimicrobial activity against various foodborne pathogens, and there are no gene families that correlate with human pathogenicity detected in the *B*. *velezensis genome*^43,44^. Therefore, we presume that the *B*. *velezensis* strain, A2, has potential as a feed supplement to detoxify ZEN.

In the current study, the doses of ZEN were selected based on a toxicity test in mice in a previous study (40 mg/kg-8% of LD50)^13^. The A2 strain was cultured in LB for 24 h because that is the optimal logarithmic growth phase for bacterial activity. Our results indicate that oral administration of a dose of 0.2 mL of A2 fermentation broth (containing the A2 strain and its secretions) exerted protective effects on ZEN-induced kidney damage in mice.

The serum activities of BUN, UA, and CRE function as biochemical markers for kidney damage. The activities of MDA, T-SOD, and GSH-PX can serve as biochemical markers for oxidative damage. Previous studies have shown that and the presence of ZEN in a diet can cause a change in the content and enzyme activity of these biochemical markers^20,21,45^. In our study, we found that ZEN induced oxidative damage in kidneys, which is in accordance with previous reports^19^. Additionally, co-administration of A2 fermentation significantly changed the serum enzyme and tissue enzymology induced by ZEN in mice. These results indicate that the A2 fermentation broth can prevent ZEN-induced kidney injury in mice.

Previous studies have shown that the ER stress pathway has been reported to participate in oxidative damage and apoptosis induced by ZEN^22–24,46^. To provide further evidence that the A2 fermentation broth exerts a protective effect from ZEN-induced kidney injury, we explored whether the A2 fermentation broth could decrease both ER stress and apoptosis induced by ZEN.

It has previously been reported that ER stress compromises the protein folding properties of the endoplasmic reticulum, which give rise to accumulation of unfolded proteins in the ER lumen. This causes the regulatory factor Bip (GRP78) to dissociate from PERK, ATF-6, and IRE1, which regulate downstream transcriptional factors that trigger apoptosis^23,24,47–49^. Apoptosis proceeds primarily through ER stress-induced activation of the transcription factors CHOP, Bax, JNK, and Caspase-12^50–52^. In the present study, our data demonstrated similar results. Multiple reports have shown that treatment with ZEN also induces ER stress in multiple cell systems^22–24,53^. This suggests that ER stress is a key mediator of damaging mechanisms induced by ZEN. The transcription factor CHOP is a key factor in ER stress-induced apoptosis, and it is up-regulated by the three branches of the unfolded protein response: PERK, ATF-6, IRE1^49,51,54^. CHOP down-regulates the anti-apoptotic protein Bcl-2 and up-regulates the pro-apoptotic protein Bax, which is necessary for Bax-mediated apoptosis^24,55^. Concurrently, we found that the same regulatory mechanisms are utilized in ZEN-induced apoptosis by regulating the Bcl-2 family proteins, which can up-regulate Bax and down-regulate Bcl-2 expression^56,57^. Meanwhile, ER stress activates IRE1, and IRE1 causes a cascade of phosphorylation incidents that ultimately activates JNK^58^. These data indicate that apoptosis induced by ZEN in the kidneys of mice is associated with the ER stress pathway.

These results confirm that ZEN-induced oxidative damage and apoptosis are associated with ER stress^59,60^. Thus, we examined whether the *B*. *velezensis* A2 strain could cope with ZEN-induced ER stress and apoptosis. Previous studies in RAW 264.7 macrophages have shown that 4-phenylbutyrate significantly decreases the ZEN-induced up-regulation of GRP78 and CHOP^24^. We found that oral administration of the A2 fermentation broth demonstrated the same regulatory mechanism. Our data also suggests that the A2 fermentation broth greatly inhibited ER stress and apoptosis induced by ZEN, as indicated by the reduction in ZEN-induced up-regulation of GRP78, CHOP, JNK, Caspase-12, and Bax in the kidneys of mice. We speculate that the detoxification mechanism of the A2 strain is similar to that of a previously reported biological antidote^27,29–31^, It may be due to the complete degradation of ZEN by the fermentation broth of the A2 strain in mice. Furthermore, the *B*. *velezensis* A2 strain did not cause kidney pathology. Its degradation product is non-toxic, and it does not cause damage to the body. This suggests that the A2 strain, like other ZEN detoxifying strains^25,27–30^, has potential as a probiotic for the practical production practices of ZEN detoxification.

In summary, we have shown that ZEN-induced kidney tissue damage was associated with ER stress induced by ZEN in cells^22^. We also demonstrate that co-treatment with fermentation broth from the *B*. *velezensis* A2 strain protects kidney cells from ZEN poisoning by inhibiting ROS generation and ER stress, suggesting that *B*. *velezensis* A2 fermentation broth treatment might be advantageous in preventing the toxicity of ZEN.

## Conclusion

This study is the first to report that treatment with *B*. *velezensis* A2 can effectively relieve the ZEN-induced oxidative damage and apoptosis in murine kidney. This study lays a foundation for the practical application of fermentation broth from the A2 strain in the future.

## Materials and Methods

### Soil, chemicals, and media

Microorganisms capable of degrading ZEN were isolated from soil samples that were collected from a farm at Shenyang in Liaoning, China, which had frequent *Fusarium* head blight epidemics. The standard chemicals for ZEN were purchased from Sigma (St Louis, MO, USA). The microbial culture was screened using minimal medium (MM) supplemented with ZEN as a carbon source 27. Luria-Bertani (LB) agar plates were used to isolate single colonies. LB and GAM (*Hopebio*, Qingdao, China) broth were used to culture the bacteria.

The MM stock solution (1/2 liter: 2.5 g NH4Cl, 1.25 g NaCl, 7.5 g KH2PO4, and 21.25 g Na2HPO4.2H2O) was adjusted to pH 7.0 using 4 M NaOH. The solution was autoclaved at 121 °C for 20 minutes, and after cooling, it was stored at room temperature.

The LB stock solution (1/2 liter: 5 g NaCl, 2.5 g yeast extract, and 5 g tryptone) was adjusted to pH 7.0 using 4 M NaOH. It was autoclaved at 121 °C for 20 minutes. After cooling, the flask is swirled to ensure mixing. For production of a solid medium, 15 g/L of agar powder was added.

### Isolation of bacterial strains able to degrade ZEN

Soil samples (2.5 g) were blended in normal saline (25 mL), and after the soil settled, 300 μL of the resulting supernatants were incubated in 30 mL of MM supplemented with ZEN (2.79 μg/mL). The cultures were incubated at 37 °C for 3 days with shaking at 150 rpm. Then, 30 mL of LB broth containing 2.79 μg/mL of ZEN was inoculated with 1% (v/v) of each culture, and this was followed by 3 days of incubation under the same conditions. This procedure was repeated 5 times. Then, each culture was centrifuged at 17,000 × g for 10 min at 4 °C. The supernatants were collected and mixed with equal volumes of chromatographic pure methanol. For both treatment groups, all solutions prepared for HPLC were filtered through a 0.22 μm-pore size filter (Millipore) before use. The dominant bacteria were streaked on LB agar plates and incubated at 37 °C for 48 h. Single colonies were inoculated into 5 mL LB medium (ZEN 7.45 μg/mL) and incubated at 37 °C (150 rpm) for 3 days. The ZEN concentrations in the samples were analyzed by HPLC. The same treatment was done for those cultured in the GAM medium. Strains with ZEN-degrading activity were stored in 25% glycerol at −80 °C until use.

### High-performance liquid chromatography (HPLC)

Analysis of ZEN toxin content of the samples by HPLC, as previously described^28,39^. The supernatant from the 0.5 mL centrifuged sample was tested, and it was added to an equal volume of chromatographic grade methanol. After 20 minutes, the reaction was complete, and it was then filtered with a 0.22 μm organic filter. Then, 20 μL was injected into the HPLC system to analyze the content of ZEN. The control group underwent the same treatment. The specific conditions of HPLC detection were as follows: Agilent ZORBAX SB-C18 (4.6 mm × 250 mm, 5 μm); Sample size: 20 μL; Mobile phase: methanol/water (80/20); Flow rate: 1.0 mL/min; Fluorescence detector and excitation wavelength: 274 nm. The percentage of ZEN removal was calculated using the following formula: 1- (peak area of ZEN in the supernatant /peak area of ZEN in the positive control) × 100%.

### Phenotypic characterization of the optimal bacteria

The A2 strain was subjected to Gram staining, morphological analysis, and 16 S rDNA sequence analysis. Genomic DNA of the A2 strain was isolated using the Ezup Column Bacteria Genomic DNA Purification Kit (B518255, Sangon, China). The standard 16 S rRNA gene primers (27 f: 5′- AGAGTTTGATCMTGGCTCAG -3′ and 1492r: 5′- CGGTTACCTTGTTACGACTT -3′) for polymerase chain reaction (PCR) were used to amplify the gene sequence of the A2 strain. The PCR cycles were as follows: 5 min of hot-start at 95 °C, followed by 35 cycles of 95 °C (30 s), 65 °C (30 s), and 72 °C (45 s), and the final 72 °C degrees to extend for 10 min). The resultant PCR product was then sequenced by Sangon (Shanghai, China). Sequences similar to the A2 gene were identified using BLAST (http://www.ncbi.nlm.nih.gov). All similar sequences were retrieved from NCBI, and a phylogenetic tree was constructed by DNAStar.

### Animals

Female Kunming mice (25 ± 2 g and 4 weeks-old) were purchased from Changchun biotechnology of China. The mice were maintained under SPF conditions with restricted-access with a humidity of 45–55%, they were maintained in 12 h light/dark cycles at a temperature of 23 ± 2 °C. Before the experiment began, the mice were given an acclimatization period of 1 week. These experiments were performed in accordance with the European Communities Council Directive of 24 November 1986 (86/609/EEC) and the principles of SPF laboratory animal care. Furthermore, the study was approved by the Ethics Committee of Shenyang Agricultural University, Shenyang, China.

### Experimental design and treatment

A stock solution of ZEN at 48 mg/mL was prepared in pure alcohol and stored at −20 °C. The working solution was prepared by diluting the stock solution into LB media and the A2 strains LB-fermentation broth.

The A2 strains were streaked on LB agar plates and incubated at 37 °C for 24 h. Single colonies were inoculated into 5 mL aliquots of LB medium and incubated at 37 °C (150 rpm) for 24 h. Then, 30 mL LB media was inoculated with 300 μL of a 24 h bacterial culture of the A2 strains and incubated at 37 °C (150 rpm) for 24 h.

The mice were divided into four groups of 10 mice per group: K group, ZEN group, A2 group, and A2 + ZEN group. Animals in each of the different treatment groups were treated daily by oral gavage (without anesthesia) for 28 days. The mice in the control group were orally administrated 0.2 mL of LB media every day. The mice in the ZEN group were orally administrated 0.2 mL of LB media containing a 40 mg/kg dose of ZEN every day. The concentration of ZEN was selected based on preliminary experiments by Long Miao *et al*.^13^. The mice in the A2 group were orally administrated 0.2 mL of the A2 LB-fermentation solution. The mice in the A2 + ZEN group were orally administrated 0.2 mL of the A2 LB-fermentation solution containing a 40 mg/kg dose of ZEN.

Twenty-four hours after administration of the final treatment, mice were sacrificed under anesthesia. Blood samples were collected, and serum was isolated. Kidney tissues were collected in cryogenic vials and stored at −80 °C until further use.

### Kidney organ indexes and Hematoxylin-Eosin (HE) staining

Twenty-four hours after the last treatment administration, the body weights of all mice were measured. Then, the mice were sacrificed, and the kidneys were excised from each mouse. Organ coefficients were calculated as the organ wet weight percentage of the total body weight. Pathological changes in kidneys tissues from 3 randomly selected mice from each group were examined using HE staining (Servicebio, Wuhan China).

### Biochemical assays

Blood samples were centrifuged at 4000 rpm for 10 min. The serum was collected and used to determine the following biochemical parameters blood urea nitrogen (BUN), uric acid (UA), and creatinine (CRE). A suspension of kidney tissue was centrifuged at 4000 rpm for 10 min. The supernatant was collected, and the activities of T-SOD, GSH-PX, and MDA were assayed. Each measurement was determined following the manufacturer’s instructions for the commercial kits obtained from the Institute of Biological Engineering of Nanjing Jiancheng, China.

### Gene expression

Total RNA was extracted from the kidney using the RNAiso Plus reagent (9108, Applied TaKaRa, Da Lian, China). Measurement of the purity and concentration of total RNA was done using a micro-volume spectrometer (uLITE, Biochrom Ltd, England) at an absorbance ratio of 260/280 nm. Values in the range of 1.8–2.0 indicate a pure RNA samples. Then, the extracted total RNA was reverse-transcribed to cDNA using a TaKaRa PrimeScript ^TM^ RT reagent kit (RR047A, Applied TaKaRa, DaLian, China).

An ABI 7500 real-time PCR system and the SYBR® Premix Ex TaqTM II kit (RR820A, Applied TaKaRa, DaLian, China) were used to conduct real-time PCR. For quantitative real time polymerase chain reaction (qRT-PCR), the total volume of the PCR reaction mixture was 20 µL, and it consisted of 2 μL of cDNA product, 0.8 μL of reverse primers, 0.8 μL of forward primers, 10 μL of Taq MasterMix solution, 6 μL of RNase-free water, and 0.4 μL of Rox. The conditions of PCR were as follows: an initial denaturation step at 95 °C for 30 s, followed by 40 cycles of 95 °C for 5 s, 60 °C for 34 s, 95 °C for 15 s, 60 °C for 1 min, and 95 °C for 15 s. Each sample was measured in triplicate. The relative changes in mRNA were calculated using the 2^−ΔΔCt^ method. The primers, shown in Table 1, were designed and synthesized by Sangon (Shanghai, China).

**Table 1:** Primers for RT-PCR analysis.

Gene	Accession No.	Primer Sequences (5′ - 3′)	Product Size/bp
*β-actin*	BC_138614.1	Forward: CTGTCCCTGTATGCCTCTG Reverse: TTGATGTCACGCACGATT	221 bp
*GRP78*	NM_001163434.1	Forward: CGCTGGGCATCATTGAAGTAA Reverse: GAGGTGGGCAAACCAAGACAT	145 bp
*JNK*	NM_001310452.1	Forward: TCCTCCAAATCCATTACCTCC Reverse: CTCCAGCACCCATACATCAAC	149 bp
*Caspase-12*	NM_009808.4	Forward: CTCAATAGTGGGCATCTGGGT Reverse: GAAGGTAGGCAAGACTGGTTC	151 bp
*Bcl-2*	NM_009741.5	Forward: CTCAGGCTGGAAGGAGAAGAT Reverse: AAGCTGTCACAGAGGGGCTAC	156 bp
*Bax*	NM_007527.3	Forward: GCAAAGTAGAAGAGGGCAACC Reverse: ACTGGACAGCAATATGGAGCT	156 bp
*CHOP*	NM_001290183.1	Forward: TTCTCCTTCATGCGTTGCTTC Reverse: AAAACCTTCACTACTCTTGACCCTG	218 bp

### Western blot analysis

The total protein form kidney tissue was obtained using the ProteinExt® Mammalian Total Protein Extraction Kit (DE101, TransGen Biotech. Beijing, China).The Easy II Protein Quantitative Kit was used to determine the protein concentrations (DQ111, TransGen Biotech. Beijing, China). The renal proteins were separated by SDS-polyacrylamide gel electrophoresis and transferred to PVDF membranes (Solarbio, Beijing, China). The membranes were incubated overnight at 4 °C with the following antibodies: β-Actin (13E5, CST, USA), GRP78 (C50B12, CST, USA), JNK (CST, USA), P-JNK (81E11, CST, USA), Caspase-12 (CST, USA), Bcl-2 (D17C4, CST, USA), Bax (D3R2M, CST, USA), CHOP (D46F1, CST, USA), and anti-DDIT3 (phosphor S30, abcam, England). Then, the membranes were washed with TBST and incubated with a secondary antibody blocking solution for 2 h at room temperature. Proteins were detected on a DNR Bio Imaging system by using the NcmECL Ultra method according to the manufacturer’s instructions (Ncmbio, Suzhou, China). The Gel Quant system was used to quantify the expression of target proteins.

### Statistical analysis

Each experiment was performed with three technical replicates. The SPSS 19.0 software was used to carry out all statistical tests, and the results are presented as mean ± standard error (X ± SE). One-way ANOVA was used to evaluate the statistical significance of the mean differences among groups. Differences were considered significant at P < 0.05.

## Electronic supplementary material

Supplementary Figure S1
