# Supplementary material for: Bacillus velezensis A2 fermentation exerts a protective effect on renal injury induced by Zearalenone in mice
Source: Sci Rep. 2018 Sep 11;8:13646. doi: 10.1038/s41598-018-32006-z (PMC6133983; doi:10.1038/s41598-018-32006-z)
Supplement: Supplementary file 1 — Supplementary Figure S1 [file 41598_2018_32006_MOESM1_ESM.doc]

Title: *Bacillus velezensis* A2 fermentation exerts a protective effect on renal injury induced by Zearalenone in mice

Author List: Nan Wang, Peng Li, Jiawen Pan, Mingyang Wang, Miao Long, Jian Zang, Shuhua Yang

Supplementary Figure S1: Mouse renal tissue protein western blot results


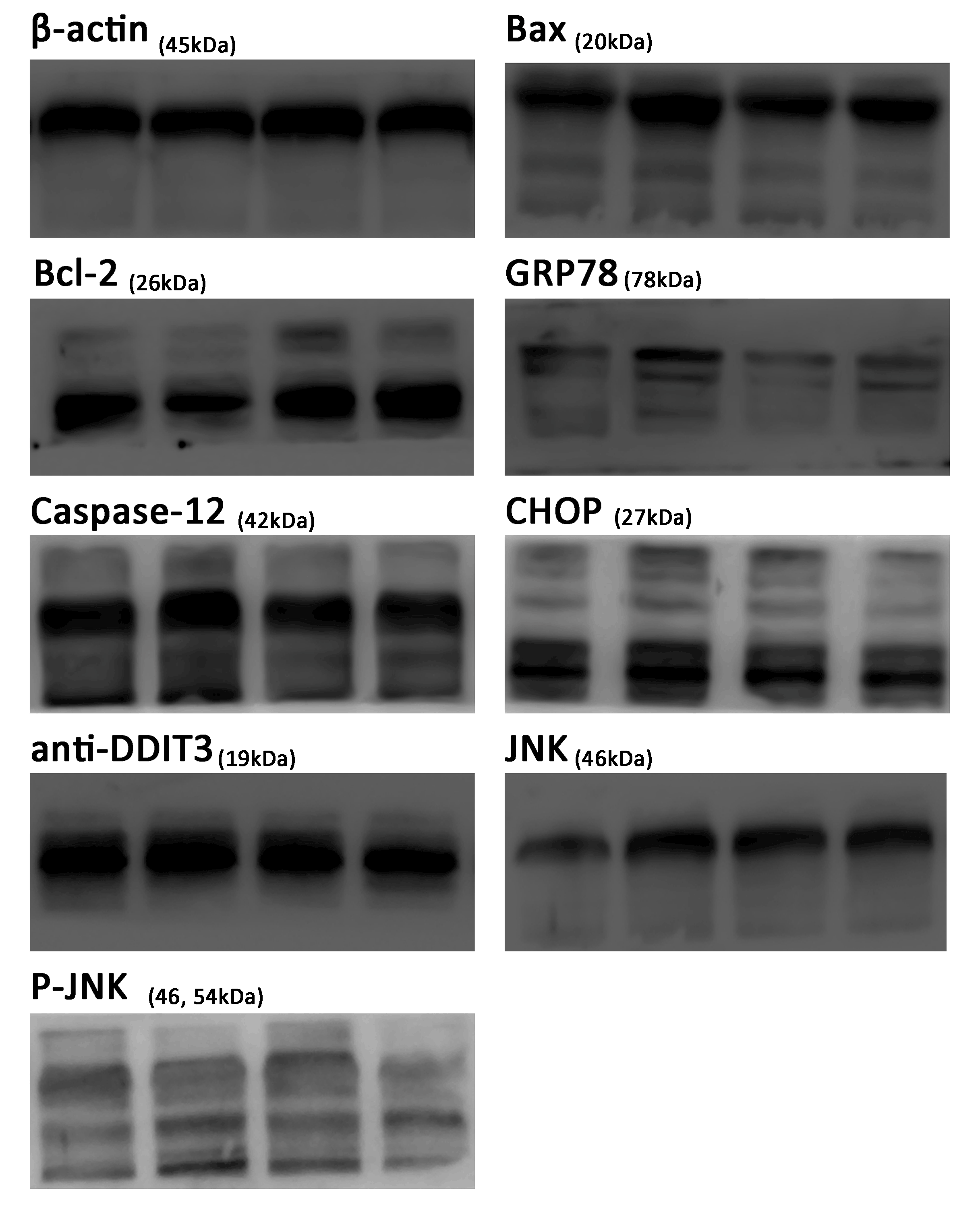


Supplementary Figure S1: protein expression associated with the ER stress. The four bands, from left to right, show control group, ZEN group, A2 group, and A2+ZEN group.
